# Immunomodulatory Effect of Flavonoids of Blueberry (*Vaccinium corymbosum* L.) Leaves via the NF-*κ*B Signal Pathway in LPS-Stimulated RAW 264.7 Cells

**DOI:** 10.1155/2017/5476903

**Published:** 2017-12-27

**Authors:** Dazhi Shi, Mengyi Xu, Mengyue Ren, Enshan Pan, Chaohua Luo, Wei Zhang, Qingfa Tang

**Affiliations:** ^1^School of Traditional Chinese Medicine, Southern Medical University, Guangzhou 510515, China; ^2^Guangdong Provincial Key Laboratory of Chinese Medicine Pharmaceutics, Southern Medical University, Guangzhou 510515, China; ^3^State Key Laboratory of Quality Research in Chinese Medicines, Macau Institute for Applied Research in Medicine and Health, Macau University of Science and Technology, Taipa, Macau

## Abstract

**Objective:**

This study aimed to explore the immunoregulatory effect of flavonoids of blueberry (*Vaccinium corymbosum* L.) leaves (FBL).

**Methods:**

The flavonoids of blueberry leaves were prepared with 70% ethanol and were identified by ultraperformance liquid chromatography/quadrupole-time-of-flight mass spectrometry (UPLC/Q-Tof-MS). The immunoregulatory effect and possible regulatory mechanisms of FBL were investigated in lipopolysaccharide- (LPS-) induced RAW 264.7 cells.

**Results:**

According to the results of UPLC/Q-Tof-MS, nine flavonoids of blueberry leaves were identified. FBL showed a significant reduction in the production of TNF-*α* in LPS-stimulated RAW 264.7 cells. FBL significantly decreased the expression of NF-*κ*B p65 and P-NF-*κ*B p65 in LPS-induced RAW 264.7 cells in a dose-dependent manner.

**Conclusion:**

Our study showed the immunoregulatory effect of FBL through the suppression of TNF-*α* via the NF-*κ*B signal pathway.

## 1. Introduction

Immunoregulation refers to the regulation of the immune system, such as stimulation, expression, amplification, or inhibition of any portion or stage of the immune response [1]. Inflammation is also part of the immunoregulation. Among various immune-related cells, macrophages not only phagocytose a variety of pathogenic microorganism, apoptotic cells, and tumor cells but also play important roles in the innate and adaptive immune responses [2]. Once infected with activation factors, macrophages release various inflammatory cytokines, including tumor necrosis factor-alpha (TNF-*α*), interleukin-1beta (IL-1*β*), and interleukin-6 (IL-6) [3]. These cytokines subsequently promote the activation and production of macrophages, then lead to macrophage infiltration and induce a local inflammatory response [4]. Many studies of the molecular mechanism of inflammation have shown that the nuclear factor-kappa B (NF-*κ*B) signal pathways [5–8], which are closely related to macrophages' growth and proliferation, play an important role in the molecular mechanism of inflammation. p65 is one of the subunits of the NF-*κ*B transcription factor protein family [9]. The heterodimers of p65 and p50 play a major role in the NF-*κ*B signal pathway. p65 is associated with DNA binding, dimerization, transcriptional activation, and nuclear translocation [9, 10]. The phosphorylation of I*κ*Bs in stimulating cells by an inducer could lead to activating the NF-*κ*B signal pathway. Then, NF-kB signal pathway activation would lead to transducing a signal from the cell surface to the nucleus [11].

Blueberry, also named cranberry, is perennial deciduous or evergreen shrubs of *Vaccinium corymbosum* L. [12]. A growing number of studies found that blueberry possesses several bioactivities such as antioxidation, free radical scavenging, anticancer, and lowering of blood pressure, plasma lipid, and blood glucose [13–16]. It has been widely used as health care products around the world. Blueberry leaves mainly contain phenylpropanoid and flavonoids [17], such as rutin, quercetin, kaempferol, and cyanidin-3-O-glu, which are similar to those of the fruits [18]. However, there are few studies about the bioactivities of blueberry leaves [8, 19].

In the present study, the chemical components of FBL were analyzed and identified by UPLC/Q-Tof-MS. Moreover, TNF-*α* was measured by ELISA, and NF-*κ*B p65 and P-NF-*κ*B p65 in LPS-induced RAW 264.7 cells were examined by Western blot. This study showed that FBL were a potential immune-adjusting reagent of the medicinal plant.

## 2. Materials and Methods

### 2.1. Chemicals and Materials

Indometacin tablets were obtained from Linfen Qilin Pharmaceutical Co. Ltd. (Shanxi, China). Dulbecco's modified Eagle's medium (DMEM) and fetal bovine serum (FBS) were obtained from Gibco (CA, USA). LPS and 3-(4,5-dimethyl-2-thiazolyl)-2,5-diphenyl-2-H-tetrazolium bromide (MTT) were purchased from Sigma-Aldrich Chemical (St. Louis, MO, USA). The enzyme-linked immunosorbent assay (ELISA) transcriptase kits for TNF-*α* were purchased from CUSABIO (Wuhan, China). The primary antibodies anti-NF-*κ*B p65 antibody and anti-P-NF-*κ*B p65 antibody were purchased from Abcam (Cambridge Science Park, UK). Peroxidase-labeled rabbit anti-mouse, sheep anti-rabbit immunoglobulin, and an enhanced chemiluminescence (ECL) detection system were obtained from Amersham (Arlington Heights, IL, USA). All other chemicals were purchased from Sigma-Aldrich Chemical. The dried leaves of blueberry were purchased from Jiangsu, China.

### 2.2. Preparation of FBL

The dried leaves of blueberry (20 kg) were first extracted for three times with 95% ethanol. The obtained extract was separated by macroporous resin D101. The 70% ethanol product was prepared into lyophilized FBL powder (17.0 g).

### 2.3. UPLC/Q-Tof-MS Analysis of FBL

Lyophilized FBL powder (0.01 g) was dissolved in 1 mL 3% DMSO (*v*/*v*). The FBL solution was solubilized by ultrasonic treatment for 5 min. Then, it was centrifuged at 15,000 rpm for 10 min. The supernatant was filtered through a 0.22 *μ*m syringe filter to be the stock solution of FBL. 50 *μ*L of the stock solution was transferred to a 1.5 mL tube, and 950 *μ*L of distilled water was added. The resulting solution was injected into the UPLC system for qualitative analysis.

The FBL sample was analyzed with the Waters Xevo G2 Q-Tof/UPLC system. Chromatographic separation was performed on a reversed-phase stationary phase (Agilent ZORBAX SB-Aq C18 column: 100 mm × 2.1 mm, 3.5 *μ*m) with an injection volume of 3 *μ*L. The mobile phase consisted of acetonitrile (A) and 0.1% formic acid (B) with a flow rate of 0.4 mL/min at 30°C. The gradient program was set as follows: 15% to 25% A at 0.0–20.0 min and 25% to 37% A at 20.0–22.0 min. The interface between UPLC and Q-Tof was the ESI source with the electrospray inlet operated in the negative mode. The column effluent was introduced into Q-Tof. Detection of the ions was performed in the full-scan mode. Data acquisition range (*m*/*z*) is 150 to 1000. Ion source parameters were as follows: capillary voltage: 3200 V, cone hole voltage: 30 V, ion source temperature: 100°C, desolvent temperature: 350°C, volume flow rate of atomization gas (N_2_): 60 L/h, volume flow rate of desolvent gas (N_2_): 600 L/h, and collision energy (CE): 20 to 50 V. The analytical data were processed by the Masslynx software.

### 2.4. Cell Lines and Cell Culture

The murine macrophage cell line RAW 264.7 cells were obtained from the Institute of Biochemistry and Cell Biology (Shanghai, China). Cells were cultured in DMEM with 10% FBS, penicillin (100 U/mL), and streptomycin (100 *μ*g/mL) in a humidified atmosphere with 5% CO_2_ at 37°C.

### 2.5. Determination of Cell Viability

Cell viability was measured by the MTT assay. RAW 264.7 cells were seeded at a density of 5 × 10^5^ cells/mL in 96-well plates. Then, various concentrations of test samples were treated for 3 h, and cells were continued to stimulate with 1 *μ*g/mL LPS for 24 h. Subsequently, MTT solution was given a final concentration of 0.5 mg/mL with incubation for 4 h at 37°C. After formazan was fully dissolved in DMSO, the absorption values were measured at 570 nm (reference, 630 nm) on a microplate reader. The cell viability in the control group (cells were treated by LPS) was set as 100%.

### 2.6. Determination of FBL Treatment Duration

RAW 264.7 cells were seeded at a density of 5 × 10^5^ cells/mL in 96-well plates. Then, cells were divided into 4 groups which were respectively treated with 62.50 *μ*g/mL FBL solution for 3 h, 6 h, 12 h, and 24 h. Cells were continued to stimulate with 1 *μ*g/mL LPS for 24 h. Cell viability was measured by the MTT assay as mentioned in Section 2.5. The control group (treated without FBL and LPS) and model group (only treated with LPS) were treated in parallel.

### 2.7. Determination of TNF-*α* by ELISA

RAW 264.7 cells were divided into the positive group, high-dose FBL group, middle-dose FBL group, and low-dose FBL group, which were treated with 44.70 mg/mL indometacin solution and 62.50, 15.62, and 3.91 *μ*g/mL FBL solution dissolved in DMEM for 6 h, respectively, and continued to stimulate with 1 *μ*g/mL LPS for 24 h. RAW 264.7 cells in the model group were just stimulated with 1 *μ*g/mL LPS for 24 h without the 6 h of pretreatment. RAW 264.7 cells in the control group were treated with neither LPS nor FBL. The supernatant was collected and mixed with the same volume of Griess reagent for 15 minutes at room temperature in the dark. The level of TNF-*α* in cultured media was determined by selective ELISA kits according to the manufacturer's instructions. The absorbance was detected on a microplate reader.

### 2.8. Western Blot Analysis

The macrophages in the above six groups were collected and resuspended in RIPA lysis buffer for 20 min at 4°C. The protein concentrations of NF-*κ*B p65 and P-NF-*κ*B p65 in the cell lysate were determined using a DC Protein Assay. Equal amounts of protein from each sample were separated by sodium dodecyl sulfate polyacrylamide gel electrophoresis (SDS-PAGE) at 80 V for 30 min and then at 120 V for 90 min and transferred onto the nitrocellulose membrane. The membranes were then incubated overnight at 4°C with the corresponding primary antibodies, followed by incubation with the appropriate secondary antibodies conjugated to horseradish peroxidase at room temperature for 2 hours. The ECL detection system was used to monitor the immune-reactive bands.

### 2.9. Statistical Analysis

All data are presented as mean ± SD. The data were analyzed by the unpaired *t*-test. All statistical analyses were performed using SPSS 16.0 software (SPSS, Chicago, IL, USA), and a value of *P* < 0.05 was accepted as statistically significant. All figures reflect the data obtained from at least three independent experiments.

## 3. Results

### 3.1. Identification of the Main Constituents in FBL

**Figure 1:**
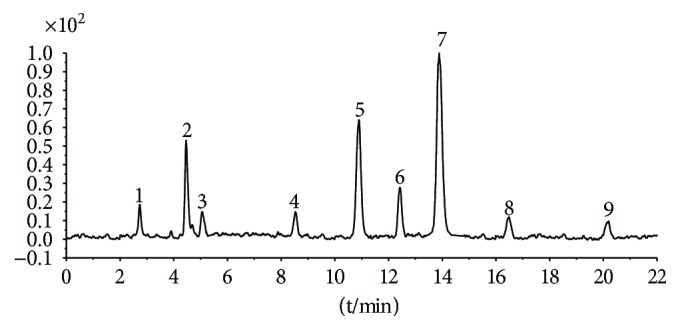
Total ion chromatogram of FBL in a negative mode. Peak attribution: myricetin (1), rutin (2), myricetin-3′-rhamnoside (3), toxicarolisoflavone (4), 3,3′,4′,5,7-pentahydroxy-6-(4-hydroxybenzyl)-flavanone (5), iridin (6), quercetin (7), cyanidin-3-(6′-malonylglucoside) (8), and kaempferol (9).

The major components of FBL were perfectly analyzed by a developed chromatographic method. The total ion chromatogram of FBL detected with UPLC/Q-Tof-MS in a negative mode was shown in Figure 1. The nine main constituents were detected as shown in Table 1. Nine peaks were identified as myricetin (1), rutin (2), myricetin-3′-rhamnoside (3), toxicarolisoflavone (4), 3,3′,4′,5,7-pentahydroxy-6-(4-hydroxybenzyl)-flavanone (5), iridin (6), quercetin (7), cyanidin-3-(6′-malonylglucoside) (8), and kaempferol (9), respectively.

### 3.2. Effects of FBL on Cell Viability

The viability of RAW 264.7 cells treated with FBL solutions was shown in Table 2. The results showed that the cell viability decreased significantly at 125.00 *μ*g/mL and higher FBL concentrations (*P* < 0.01), but there was no difference among 62.50, 31.25, 15.63, 7.81, and 3.91 *μ*g/mL groups. Therefore, 62.50 *μ*g/mL FBL was set as the high-dose group and then 15.63 and 3.91 *μ*g/mL FBL as the middle-dose and low-dose groups, respectively.

### 3.3. FBL Treatment Duration of RAW 264.7 Cells

**Figure 2:**
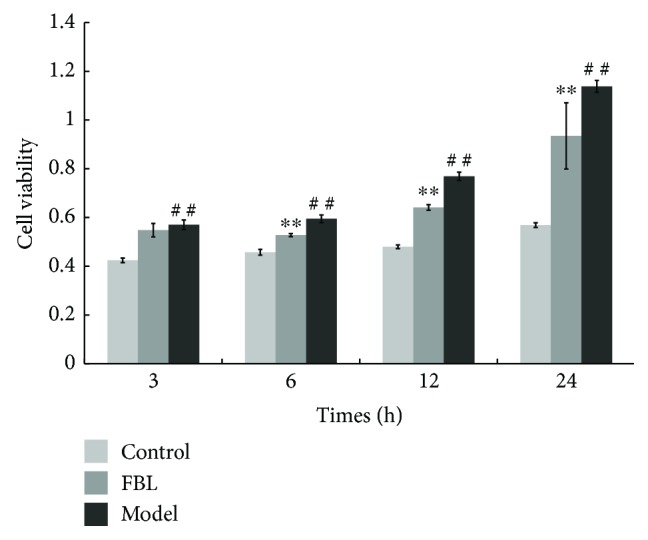
FBL treatment duration of RAW 264.7 cells. Control groups were treated without LPS and FBL, model groups were treated with only 1 *μ*g/mL LPS, and FBL groups were treated with 1 *μ*g/mL LPS and 62.50 *μ*g/mL FBL. ^∗∗^*P* < 0.01: FBL group versus model group during the same treating time. ^##^*P* < 0.01: model group versus control group during the same treating time.

Cell viability was determined via the MTT assay (Figure 2). The cell viability of model groups compared with control groups of different FBL treatment durations significantly increased (*P* < 0.01). However, the cell viability of the FBL group compared with model groups decreased significantly among 6, 12, and 24 h FBL treatment duration groups (*P* < 0.01). These data indicated RAW 264.7 cells treated with FBL for 6 h inhibited cell proliferation effectively.

### 3.4. Effects of FBL on TNF-*α* Levels

**Figure 3:**
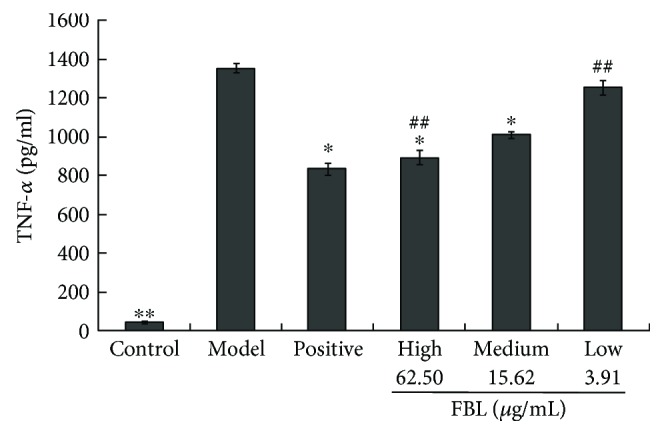
Effects of FBL on TNF-*α* levels in LPS-stimulated RAW 264.7 cells. Control group treated without LPS and FBL; model group treated with only 1 *μ*g/mL LPS for 24 h; positive group treated with 44.70 mg/mL indometacin for 6 h and 1 *μ*g/mL LPS for 24 h; and FBL groups treated with 62.50, 15.62, and 3.91 *μ*g/mL FBL for 6 h, respectively, and 1 *μ*g/mL LPS for 24 h. ^∗^*P* < 0.05 and ^∗∗^*P* < 0.01 versus model group. ^##^*P* < 0.01 versus medium-dose FBL group.

The levels of TNF-*α* in the culture media were measured by ELISA. As presented in Figure 3, the concentrations of TNF-*α* in the model group were increased significantly compared with those in the control group (*P* < 0.01). The levels of TNF-*α* in the positive group were significantly decreased compared to those in the model group (*P* < 0.05). The levels of TNF-*α* in the medium-dose FBL and high-dose FBL groups were significantly decreased compared to those in the model group (*P* < 0.05). The levels of TNF-*α* in the high-dose FBL and low-dose FBL groups were significantly different compared to those in the medium-dose FBL group (*P* < 0.01). These data indicated FBL negatively regulated the production of TNF-*α* at the transcriptional and translational levels in LPS-induced RAW 264.7 cells in a concentration-dependent manner.

### 3.5. Effects of FBL on the Expression of NF-*κ*B p65 and P-NF-*κ*B p65

**Figure 4:**
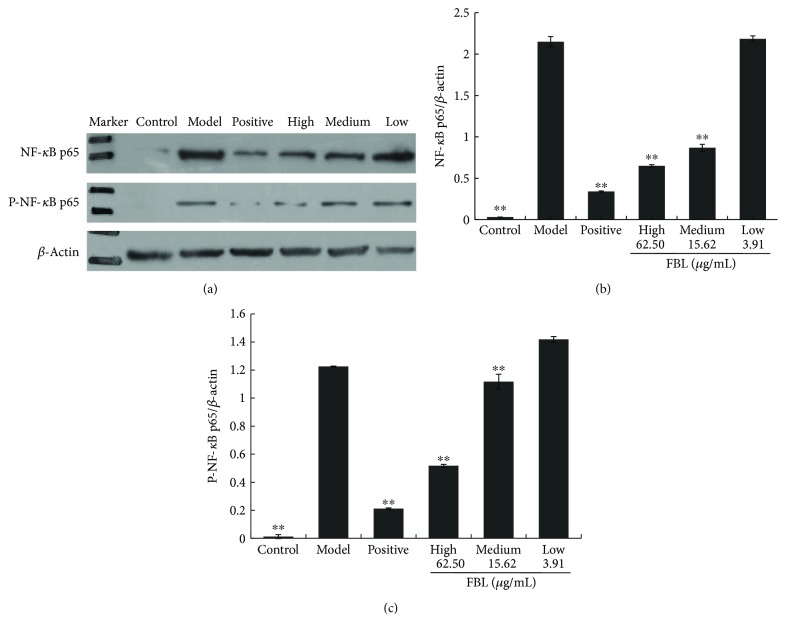
Effects of FBL on the expressions of NF-*κ*B p65 and P-NF-*κ*B p65 in LPS-stimulated RAW 264.7 cells. Control group treated without LPS and FBL; model group treated with only 1 *μ*g/mL LPS for 24 h; positive group treated with 44.70 mg/mL indometacin for 6 h and 1 *μ*g/mL LPS for 24 h; and FBL groups treated with 62.50, 15.62, and 3.91 *μ*g/mL FBL for 6 h, respectively, and 1 *μ*g/mL LPS for 24 h. (a) The picture of NF-*κ*B p65, P-NF-*κ*B p65, and *β*-actin; (b) the ratio of NF-*κ*B p65 to *β*-actin; and (c) the ratio of P-NF-*κ*B p65 to *β*-actin. ^∗∗^*P* < 0.01 versus model group.

To identify whether FBL mediates its anti-inflammatory activities by modulating NF-*κ*B activation, the expression of NF-*κ*B p65 and P-NF-*κ*B p65 in LPS-activated macrophages treated with FBL was examined by Western blot (Figure 4). The level of NF-*κ*B p65 and P-NF-*κ*B p65 proteins in the model group was increased significantly compared with that in the control group (*P* < 0.01). The level of NF-*κ*B p65 and P-NF-*κ*B p65 proteins of the positive group was decreased significantly compared to that in the model group (*P* < 0.01). The concentrations of NF-*κ*B p65 and P-NF-*κ*B p65 of the middle- and high-dose groups were decreased significantly compared to those of the model group (*P* < 0.05). These findings indicated that the anti-inflammatory action of FBL is at least partially due to the inhibition of NF-*κ*B-dependent gene transcription.

## 4. Discussion

In this experiment, different columns and mobile phase conditions were tested and the appropriate chromatographic condition was obtained. Furthermore, the Q-Tof and MS conditions were determined after several attempts. The negative mode was selected because of better graphs and data compared. The parameters capillary voltage, collision energies, and ion source temperature were determined after multiple trials. The flavonoids of blueberry leaf (FBL) extract were identified by UPLC/Q-Tof-MS, mainly containing myricetin (1), rutin (2), myricetin-3′-rhamnoside (3), toxicarolisoflavone (4), 3,3′,4′,5,7-pentahydroxy-6-(4-hydroxybenzyl)-flavanone (5), iridin (6), quercetin (7), cyanidin-3-(6′-malonylglucoside) (8), and kaempferol (9). The constituents were identified on the basis of references and mass spectrometry data, and several constituents were identified based on the reference standards.

The macrophage-based innate immune response plays an important role in congenital immune response. LPS-stimulated RAW 264.7 murine macrophage cells are generally considered a suitable model to study the immunomodulatory and anti-inflammatory effects of drugs [20]. In this study, the LPS-stimulated RAW 264.7 cell was prepared as an inflammation model. Not only the levels of TNF-*α* but also those of IL-6 were determined. After being stimulated with LPS, RAW 264.7 cells induced and released inflammatory factors IL-6 and TNF-*α* rapidly. The level of IL-6 showed no difference among the three FBL dose groups compared to the model group. This illustrated that FBL had no effects on IL-6, which is related to the STAT signal pathway [21]. The results of TNF-*α* indicated that FBL could regulate in a concentration-dependent manner. Referenced to the studies of the NF-*κ*B signal pathway, the production of cytokine TNF-*α* could activate the NF-*κ*B signal pathway. Thus, the following experiment was designed to focus on the NF-*κ*B signal pathway.

It has been reported that the nuclear transcription factor NF-*κ*B is involved in the transcriptional regulation of a variety of cytokines and inflammatory mediators [22], which can specifically bind to a specific site of gene promoters and enhancer sequences to promote transcription and expression [23]. NF-*κ*B p65 located in the cytoplasm would be inactivated after binding to its inhibitor I*κ*B [24]. When it is subjected to external stimuli (such as LPS), I*κ*B*α* phosphorylates, degrades, and dissociates from the NF-*κ*B/I*κ*B*α* complex [25, 26]. The activated NF-*κ*B p65 following the translocation into the cell nucleus combines with the target gene promoter or enhancer [27]. It continued to rapidly induce the synthesis and transcription of target gene mRNA. In our study, the level of NF-*κ*B pathway protein p65 and its phosphorylated protein P-p65 was decreased in the high- and middle-dose groups compared to the model group. The result indicated that the FBL could inhibit the transcription of the NF-*κ*B-dependent gene. It also illustrated that the anti-inflammatory of FBL is partially caused by inhibition of NF-*κ*B-dependent gene transcription.

## 5. Conclusion

In summary, nine flavonoids of blueberry leaves were identified by UPLC/Q-Tof-MS which are similar to those of the blueberry fruit. The FBL could significantly reduce the expression and release of TNF-*α* in LPS-induced RAW 264.7 cells in a dose-dependent manner. Furthermore, the results of p65 and P-p65 suggested that FBL inhibits inflammation-related factor expression by suppressing the NF-*κ*B signal pathway. Our findings provided some reference for the further development and utilization of the blueberry plant.

## Figures and Tables

**Table 1:** Identification of nine constituents of FBL by UPLC/Q-TOF-MS.

Number	R_T_/min	Constituents	Elemental composition	MS^1^/MS^2^ (*m*/*z*)	Chemical structures
1	2.7	Myricetin	C_15_H_10_O_8_	317.0391 [M-H]^−^ 179.0031, 151.0121, 109.0338	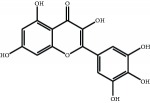
2	4.5	Rutin	C_27_H_30_O_16_	609.1937 [M-H]^−^ 301.0345, 300.0273	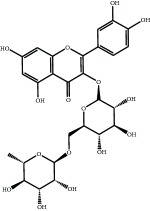
3	5.2	Myricetin-3′-rhamnoside	C_21_H_20_O_12_	463.0771 [M-H]^−^ 301.0293, 151.0093	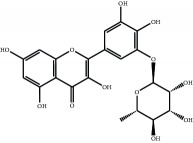
4	8.5	Toxicarolisoflavone	C_23_H_22_O_7_	409.1559 [M-H]^−^ 179.0310, 161.0223	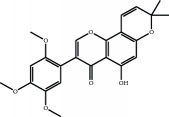
5	10.9	3,3′,4′,5,7-Pentahydroxy-6-(4-hydroxybenzyl)-flavanone	C_22_H_18_O_8_	409.1484 [M-H]^−^ 179.0357, 135.0451	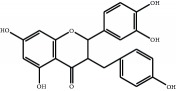
6	12.4	Iridin	C_24_H_26_O_13_	521.1553 [M-H]^−^ 359.1210, 315.1213, 297.1056, 163.0369	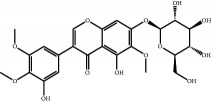
7	13.2	Quercetin	C_15_H_10_O_7_	301.0283 [M-H]^−^ 178.9975, 151.0028	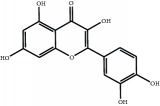
8	16.4	Cyanidin-3-(6′-malonyl-glucoside)	C_24_H_23_O_14_	533.1048 [M-H]^−^ 357.0873, 301.0362	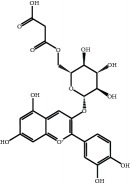
9	20.2	Kaempferol	C_15_H_10_O_6_	285.0196 [M-H]^−^ 159.0665, 93.0676	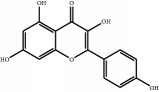

**Table 2:** Effects of FBL on the viability of RAW 264.7 cells.

FBL (*μ*g/mL)	500.00	250.00	125.00	62.50	31.25	15.63	7.81	3.91
Cell viability (%) (X ± S)	74.80 ± 4.59^∗∗^	83.15 ± 1.76^∗∗^	88.75 ± 1.78^∗∗^	101.50 ± 1.97	100.84 + 4.32	101.05 ± 3.68	100.58 ± 2.76	100.33 ± 3.45

Control groups were treated only with 1 *μ*g/mL LPS; the cell viability in the control group (cells were treated by LPS) was set as 100%. FBL groups were treated with 1 *μ*g/mL LPS and different concentrations of FBL. ^∗∗^*P* < 0.01 versus control group treated with only LPS.
